# Attach Me If You Can: Murine Norovirus Binds to Commensal Bacteria and Fungi

**DOI:** 10.3390/v12070759

**Published:** 2020-07-14

**Authors:** Jasmine L. Madrigal, Sutonuka Bhar, Samantha Hackett, Haley Engelken, Ross Joseph, Nemat O. Keyhani, Melissa K. Jones

**Affiliations:** Microbiology and Cell Science Department, IFAS, University of Florida, Gainesville, FL 32611, USA; jl.madrigal@ufl.edu (J.L.M.); sutonuka.bhar@ufl.edu (S.B.); samantha.hackett@ufl.edu (S.H.); hengelken@ufsa.ufl.edu (H.E.); josephr1@ufl.edu (R.J.); keyhani@ufl.edu (N.O.K.)

**Keywords:** norovirus, murine norovirus, virus–bacterial interaction, fungal attachment

## Abstract

The presence of commensal bacteria enhances both acute and persistent infection of murine noroviruses. For several enteric viral pathogens, mechanisms by which these bacteria enhance infection involve direct interactions between the virus and bacteria. While it has been demonstrated that human noroviruses bind to a variety of commensal bacteria, it is not known if this is also true for murine noroviruses. The goal of this study was to characterize interactions between murine noroviruses and commensal bacteria and determine the impact of bacterial growth conditions, incubation temperature and time, on murine norovirus attachment to microbes that comprise the mammalian microbiome. We show that murine noroviruses bind directly to commensal bacteria and show similar patterns of attachment as human norovirus VLPs examined under the same conditions. Furthermore, while binding levels are not impacted by the growth phase of the bacteria, they do change with time and incubation temperature. We also found that murine norovirus can bind to a commensal fungal species, *Candida* *albicans*.

## 1. Introduction

Human noroviruses (HNoVs) are the leading cause of gastrointestinal illness worldwide, responsible for 685 million infections each year [1]. In the U.S., they are responsible for approximately 20 million infections that cost the national economy $2.8 billion in health care costs and missed worker productivity [2]. In developing countries, HNoVs are estimated to cause 200,000 deaths in children under 5 years of age and are also increasingly associated with devastating infections in immunocompromised hosts [3,4,5,6]. Despite this large disease burden, relatively little is known about the pathogenesis of this virus. Investigations into mechanisms of disease have been hindered by the lack of a robust cell culture system for human noroviruses and by the host specificity of the virus, which hinders mechanistic studies within its natural, human host. Therefore, murine norovirus (MNV) is a commonly used surrogate because it can be readily grown in vitro and provides the use of a small, tractable animal model for in vivo studies. 

Commensal bacteria enhance infection of murine noroviruses whereby antibiotic depletion of the culturable gut flora results in reduced viral titers during both acute and persistent infection [7,8]. These bacteria can also enhance infection of human norovirus in vitro, in some, but not all, cell types [7,9]. The impact of commensal bacteria on acute infection of human norovirus during natural infection of the human host is not known, but select commensal bacteria do not enhance infection in off-target hosts, such as gnotobiotic piglets [10,11]. For both human and murine noroviruses, the mechanisms of bacterial enhancement of infection are still unclear [7].

Several enteric viruses, including noroviruses, can directly bind to commensal bacteria. For some viruses, this direct interaction facilitates the enhancement of infection. For example, the interactions between poliovirus and commensal bacteria enhances infection through increased viral stability, improved receptor engagement, and augmenting viral fitness through increasing viral co-infection and facilitating genetic recombination [12,13,14]. For murine noroviruses, it is unknown if direct interactions between virus and bacteria alter viral infection, but it has been shown that commensal bacteria can impact the regionalization of MNV infection within the intestine through bile acid-mediated pathways [15]. 

While the intestinal microbiome is heavily comprised of bacterial species, it also consists of viruses and commensal fungi. *Candida albicans* is a fungal species commonly isolated from the guts of healthy individuals, but can cause gastrointestinal candidiasis under immune-compromised conditions [16,17]. This fungus exhibits dimorphic growth habits, meaning that it can grow in either a single-celled (yeast-like) form or a multicellular (hyphal) form both in culture and in vivo in response to varying environmental cues including temperature, nutrient availability, and pH [18]. While hyphal forms of *C. albicans* have been shown to be associated with penetration into epithelial cells and subsequent damage to the host, yeast-like forms are largely associated with a commensal state and are assumed to make up a large proportion of non-pathogenic *C. albicans* populations in the healthy human gut [19]. To date, there are no reports of norovirus or other enteric viral pathogens interacting with commensal fungi. 

In this study, we quantified attachment of murine norovirus and human norovirus virus-like particles (VLPs) to a panel of commensal bacteria, and we also quantified murine noroviruses binding to the fungal species, *C. albicans*. We further explored MNV interactions with bacteria by examining the stability of virus–bacterial interactions over time and the effect of temperature and bacterial growth phase on viral attachment. The goal of this research was to characterize attachment of murine norovirus to commensal microbial organisms, representative of a healthy human gut and to compare the binding of murine and human noroviruses to commensal bacteria. Results showed that MNV binds to commensal bacteria in a manner similar to what has been observed for HNoVs, and is likewise capable of binding a variety of bacterial species, albeit with varying degrees of attachment. Neither bacterial growth phase nor temperature significantly altered MNV binding to any of the bacteria tested, but interactions between MNV and some bacterial species remained relatively stable over 24 h. This study is the first to demonstrate that, like human noroviruses, murine noroviruses directly bind to commensal bacteria as well as a human commensal fungus, and it provides a foundation for future investigation into mechanisms of bacterial enhancement of MNV infection.

## 2. Materials and Methods

### 2.1. Bacterial and Cell Line Growth Conditions

Bacterial isolates *Enterobacter cloacae* (ATCC 13047, ATCC, Gaithersburg, MD), *Escherichia coli* (ATCC 1129), *Pseudomonas aeruginosa* (PAO1, provided by Dr. Paul Gulig, Molecular Genetics and Microbiology Dept., University of Florida), *Lactobacillus acidophilus* (ATCC 4356, provided by Dr. Graciela Lorca, Microbiology and Cell Science Dept., University of Florida), *L. gasseri* (ATCC 3323, provided by Dr. Lorca), and *Bacteroides dorei* DSMI 7899 (provided by Dr. Eric Triplett, Dept. of Microbiology and Cell Science, University of Florida) were used in virus–bacterium attachment assays. All strains are stored in 50:50 medium:glycerol at −80 °C until use. *E. cloacae*, *E. coli*, and *P. aeruginosa* were cultivated on Luria Bertani agar (LA) and Luria Bertani broth (LB) containing 1% NaCl at 37 °C under aerobic conditions. *L. acidophilus* and *L. gasseri* were cultivated in MRS broth and agar (BD Biosciences) at 37 °C under aerobic and anaerobic conditions, respectively, using an anaerobic chamber and Anaerobic Gas (Thermo Fisher, Waltham, MA, USA).

To generate stationary phase cultures bacteria were inoculated into appropriate medium and incubated overnight at 37 °C [20]. *E. cloacae*, *E. coli*, and *P. aeruginosa* were grown with shaking (200 rpm) while *L acidophilus* and *L. gasseri* were grown statically. To generate log-phase cultures, stationary phase cultures were diluted 1:100 into 40 mL of appropriate media and were further incubated at 37 °C for 7 h. 

RAW264.7 (ATCC TIB-71, ATCC, Gaithersburg, MD) and 293T (provided by Dr. Stephanie Karst, Molecular Genetics and Microbiology Dept., University of Florida) cells used for generating recombinant MNV-1 were cultured in DMEM with 10% fetal bovine serum at 37 °C under 5% CO2. Media for all cell cultures contained 100 U/mL penicillin and 100 µg/mL streptomycin.

### 2.2. Virus Production

Recombinant Murine Norovirus-1 (MNV-1) was generated using pSPMNV-1.CW3 (provided by Dr. Stephanie Karst) as previously described [21] and used in all MNV–bacteria attachment assays. Briefly, virus stocks were made by transfecting 5 µg of infectious clone per 10^6^ 293T cells. After 24 h, cells were harvested and subjected to freeze-thawed cycles. The supernatant was collected and clarified through centrifugation. Clarified supernatant (5 mL) was used to infect T-175 flasks of RAW264.7 cells that were 90% confluent. After 36–48 h, RAW264.7 cells were subjected to three freeze-thaw cycles. Lysates were then purified through a 25% sucrose cushion and MNV-1 titers determined using a standard TCID_50_ assay [22]. Human Norovirus GII.4 VLPs were purchased from Creative Biolabs (CBS-V700, Shirley, NJ). Stock VLP was divided into 10 ug aliquots and stored at −80 °C.

### 2.3. Virus–Bacteria Attachment Assays

For all experiments, bacterial concentration was determined by OD_600_. After log or stationary phase growth, bacterial cells were washed twice with 1 × PBS and adjusted to a final concentration of 1 × 10^8^ cells/mL. For HNoV attachment assays, 1 × 10^8^ bacterial cells were inoculated with 10 µg of HNoV GII.4 VLP (Creative Biolabs, CBS-V700) as previously reported [23,24]. This amount of VLP contains approximately 2 × 10^9^ VLP particles as determined by nanosight analysis, resulting in a 20:1 ratio of VLP to bacterium. For MNV-1 assays 1 × 10^8^ bacteria were inoculated with 1 × 10^7^ TCID_50_ of virus, resulting in a ration of 0.1:1 virus to bacterium. Virus–bacteria mixtures were incubated at either 4 °C, room temperature or 37 °C with constant, gentle agitation. For time course assays virus–bacteria mixtures were incubated at 37 °C with constant, gentle rotation and at appropriate time points, an aliquot of the sample was centrifuged at 10,000× *g* for 5 min and the supernatant discarded. Cells were washed twice with equal volume of 1 × PBS to remove any unbound virus and the concentrated bacterial pellet was processed as described below. For gram-positive bacteria, total RNA was extracted using the Trizol RNA extraction method. The cell pellets were resuspended in 1 mL of Trizol reagent (Thermo Fisher, 15596018) and incubated for 5 min at room temperature; 200 µL of chloroform (Acros Organics, Fair Lawn, NJ, 15821-0010) was added to each sample and tubes were incubated on ice for 3 min. Samples were centrifuged at 12,000× *g* for 15 min at 4 °C. The upper RNA-containing aqueous layers were transferred to tubes containing 10 µg of RNAse-free glycogen (Invitrogen, Carlsbad, CA AM9510) and 500 µL of isopropanol (Fisher Scientific, BP2618-4). Samples were incubated at −20 °C for 10 min. Samples were centrifuged at 12,000× *g* for 10 min at 4 °C, the supernatant was discarded, and the pellets were washed with 1 mL of 75% EtOH. Samples were then centrifuged at 7500× *g* for 5 min at 4 °C and the supernatant was poured off. Samples were incubated with caps open for 5 min at room temperature to remove remaining EtOH. The pellet containing RNA was resuspended in 50 µL of nuclease-free water and further incubated with caps open at 60 °C for 10 min. Samples were stored at −80 °C. For gram-negative bacteria, the cell pellets were resuspended in lysis buffer and total RNA was extracted according to manufacturer’s instructions (Zymo RNA MiniPrep kit, Irvine, CA, R1055). For all attachment assays, experiments were performed a minimum of three times with two technical replicates included in each experiment. 

HNoV attachment was quantified by flow cytometry as previously described [20]. MNV attachment was quantified using RT-qPCR. RNA concentration was determined using a nanodrop and 1 µg of RNA was used to generate cDNA (Promega M-MLV, M1701). Neat cDNA was then amplified by qPCR using SYBR Green (Applied Biosystems PowerUp SYBR Green Master Mix, A25741) and previously published primers targeting a conserved region of VP1 [7]. Standard curves for quantifying MNV-1 attachment to bacteria were generated using serial dilutions of pSPMNV-1. CW3 and were included on all qPCR plates.

### 2.4. Fungal Experiments

For all experiments, fungal concentration was determined by OD_600_. *C. albicans* was inoculated into 5 mL of Sabouraud Dextrose Broth (SDB) and incubated at 25 °C overnight. Prior to conducting attachment assays, conditions were optimized to ensure growth of single-celled yeast, rather than hyphal forms of the fungus (Supplemental and Figure S1). Cultures were washed twice with 1 × PBS and adjusted to a final concentration of 1 × 10^7^ cells/mL and inoculated with 1 × 10^7^ TCID_50_ of MNV. Virus–fungi mixtures were incubated at 37 °C for 1 h, afterwards, samples were centrifuged and washed twice with an equal volume of 1 × PBS to remove the unbound virus. Total RNA was extracted using the Trizol RNA extraction method and MNV attachment was quantified using RT-qPCR, both performed as previously described.

### 2.5. Electron Microscopy

*E. cloacae* were grown overnight (~18 h) and the bacterial pellet was washed with 1 × PBS and OD_600_ was obtained. The final concentration was diluted to 1 × 10^8^ CFU/mL and a virus–bacteria attachment assay was performed as outlined above. Bacteria were incubated with either PBS as mock or MNV (0.1 MOI). After attachment, the bacteria were resuspended in fresh media and grown for 12 h. The culture was then pelleted, and the pellet was dissolved in 2% paraformaldehyde in PBS. This homogenous mixture was used for Scanning Electron Microscopy (SEM), which was performed by the ICBR Microscopy Core (University of Florida).

For sample preparation, GTTP Isopore Membrane Filters (0.2 µm, Merck Millipore, Burlington, MA, USA) were treated with a 1:10 Poly-L-lysine solution (Sigma-Aldrich, St. Louis, MO, USA) and used to filter samples. The filtrate was fixed using Trump’s fixative (EMS) and washed in 0.1 M sodium cacodylate, pH 7.24, post-fixed with buffered 2% OsO4, water-washed and dehydrated in a graded ethanol series 25% through 100% with 25% increments. Tousimis Autosamdri-815 (Tousimis, Rockville, MD) was used to point dry the samples for mounting onto a 12 mm Carbon Conductive Adhesive Tab and an aluminum stub (EMS). Mounted samples were then sputter-coated with gold/palladium using the Denton Desk V sputter coater (Denton Vacuum, Moorestown, NJ) and imaged with Hitachi SU-5000 FE-SEM (Hitachi High Technologies Dallas, TX) with the use of EM Wizard software. Attachment assays for electron microscopy experiments were performed twice with a minimum of 15 pictures captured during imaging of each experiment. Images were collected by the ICBR core facility so as to reduce selection bias. 

## 3. Results

### 3.1. Direct Attachment of Murine Norovirus to Commensal Bacteria

**Figure 1:**
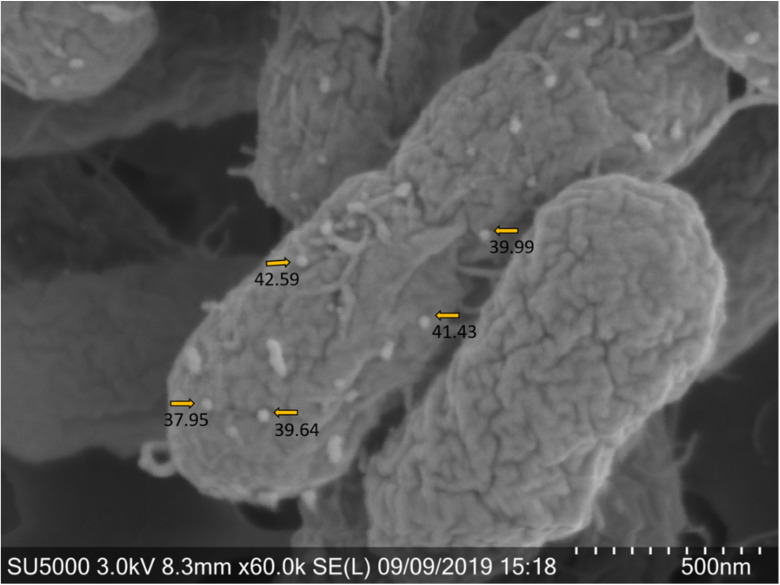
Murine norovirus (MNV) attach to various regions of the outer membrane of *Enterobacter cloacae*. Scanning Electron Microscopy (SEM) image of *E. cloacae* after attachment assay with MNV shows virus particles bound on the surface of the bacteria (representative image of *n* = 2 experiments). Arrows denote viral particles bound to the surface of bacteria as determined by particle size (in nm), which was calculated using ImageJ software.

Previously published electron microscopy images have shown HNoV binding to *E. cloacae* and demonstrated viral binding to multiple areas around the bacterial cell such as the extracellular polysaccharide matrix (EPS), the outer membrane, pili and flagella [24,25]. Thus, scanning electron microscopy was performed using MNV and *E. cloacae*. The resulting images demonstrated that this virus also binds directly to this commensal bacterium (Figure 1). However, unlike HNoVs [24], MNVs did not appear to bind to bacterial appendages, but rather were almost exclusively scattered along the outer membrane. 

**Figure 2:**
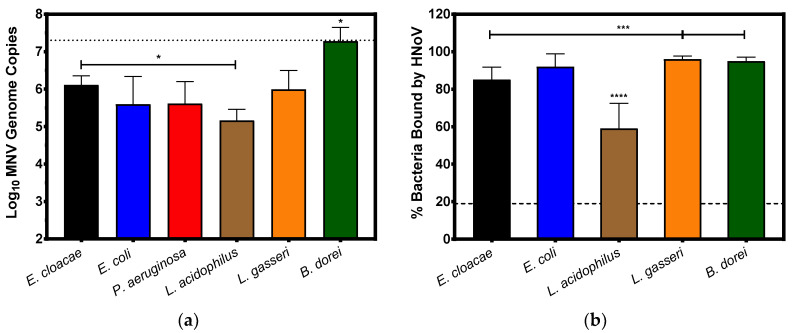
Noroviruses attach to select commensal bacteria after one hour of incubation. (**a**) MNV-1 (10^7^ TCID50/mL) was used to inoculate diluted bacterial cultures which were incubated at 37 °C for 1 h. Viral attachment was quantified using RT-PCR. MNV-1 genomes were detected in all bacterial cultures and levels of detection in *B. dorei* samples were significantly higher (*p* ≤ 0.01) than all other bacteria. Detection of MNV in *L. acidophilus* samples was only significantly lower than *E. cloacae* (* = *p* = 0.03). Dashed line indicates average amount of input virus. (**b**) HNoV VLPs (10 µg) were added to diluted bacterial cultures and incubated at 37 °C for 1 h. VLP attachment was measured using flow cytometry and percent attachment quantified using Overton Subtraction compared to isotype controls. The percent of *L. acidophilus* bacteria bound by the VLPs was significantly less (**** = *p* < 0.0001) than all other bacteria tested. Binding of *E. cloacae* populations was also significantly lower than binding to either *L. gasseri* or *B. dorei* (*** = *p* ≤ 0.005). Dashed line denotes limit of detection of the assay. (*n* = 3 for all attachment assays). Statistical analysis was performed using an unpaired Student’s *t* test in GraphPad Prism.

It has been previously demonstrated that human noroviruses bind to a variety of commensal bacteria [24], therefore, we set out to determine if murine noroviruses are also capable of this wide-spread bacterial attachment. When choosing our panel of bacteria, we selected representative species of the phyla Bacteroidetes and Firmicutes (*B. dorei* and *Lactobacillus spp*., respectively) as these are the predominant phyla of the mammalian gut. We also selected two Proteobacteria species (*E. cloacae* and *E. coli*) to assess binding to enteric bacteria as well as *P. aeruginosa* to assess binding to a non-enteric bacterium. Similar to what has been observed with HNoV [24], MNV binds to a wide variety of bacteria but to varying degrees (Figure 2a). Similar levels of binding were observed between *E. cloacae*, *E. coli*, *P. aeruginosa*, and *L. gasseri* after a 1 h incubation with MNV. Slightly lower levels of binding to *L. acidophilus* were observed compared to other bacteria but were significantly (*p* = 0.03) lower than *E. cloacae*, while the virus bound to *B. dorei* at significantly (*p* ≤ 0.01) higher levels compared to the Proteobacteria species. 

Attachment assays using HNoV GII.4 VLPs were performed using the commensal bacteria to compare binding trends between human and murine noroviruses. *P. aeruginosa* was excluded from the analysis of HNoV attachment due to technical issues with the flow cytometry read-out using this bacterium. Similar to what has been previously observed with HNoV positive stool samples [24], HNoV VLPs were capable of binding to a wide variety of commensal bacteria (Figure 2b). No significant differences in HNoV binding were observed between the Proteobacteria species (*E. coli* and *E. cloacae*); however, HNoV-bound *E. cloacae* significantly less compared to either *L. gasseri* or *B. dorei* (*p* < 0.005). The input particle ratios and methods of analysis are different between MNV and HNoV VLPs and thus absolute quantities of binding cannot be compared. However, the trends of viral attachment to the panel of bacteria do show some commonalities. As with MNV, we observed the lowest frequency of HNoV binding to *L. acidophilus* populations and binding to this bacterium was significantly lower (*p* < 0.0001) than binding to any of the other bacteria tested (Figure 2b). Since both viruses bind significantly less to *L. acidophilus* and relatively high to other bacteria, this suggests that *L. acidophilus* either lacks or does not express enough of a specific surface structure(s) necessary for high levels of viral attachment. 

### 3.2. Temporal Attachment of Murine Norovirus to Commensal Bacteria

**Figure 3:**
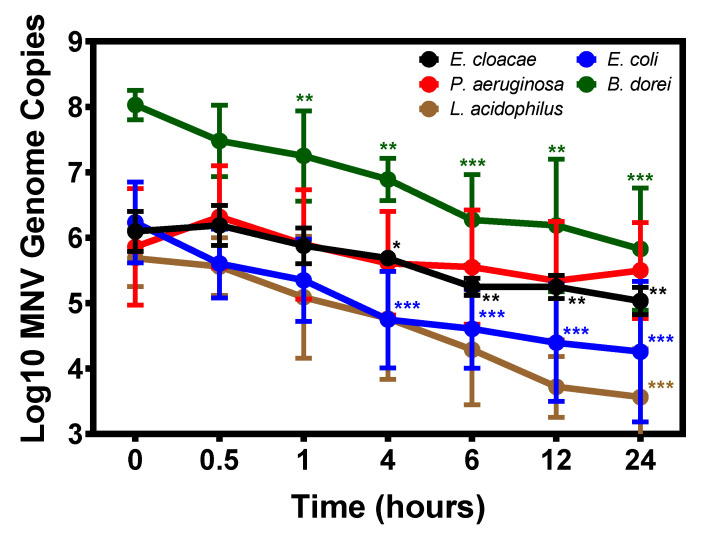
Temporal binding of MNV to commensal bacteria. MNV-1 was inoculated into diluted bacterial cultures, incubated at 37 °C and sampled at the indicated time points. Virus concentrations were determined using RT-qPCR. MNV-1 genomes were detected immediately (0 h) in all bacterial cultures and significantly decreased over 24 h for all bacteria (* = *p* ≤0.05, ** = *p* ≤0.01, *** = *p* ≤ 0.001)except *P. aeruginosa* (*n* = 3). Significance over time was determined using one-way ANOVA and differences at given time points between specific samples were analyzed using unpaired Students *t* test, both in GraphPad Prism.

All currently published data investigating norovirus attachment to commensal bacteria focuses on a single time point [23,24,26,27], and we questioned whether binding would change with shorter or longer incubation periods. To investigate this, we used RT-q-PCR analysis to quantify MNV binding to our panel of commensal bacteria from 0 to 24 h of incubation. Results showed that MNV binds to all of the bacteria almost immediately, as evidenced by the detection of viral RNA at the 0 h time point (Figure 3). When examining attachment to Proteobacteria, viral attachment to *E. cloacae* and *P. aeruginosa* shows slow declines in viral attachment after 0.5 h. Viral attachment to *P. aeruginosa* do not significantly differ from time 0 h attachment at any point of the tested time points, but significant decreases in detected viral genome copies in *E. cloacae* samples were observed beginning at 4 h. Conversely, while initial binding to *E. coli* is similar to the other bacteria tested, viral attachment to this strain declines immediately and levels off after 4 h of incubation. 

MNV appears to display similar binding kinetics to both *B. dorei* and *L. acidophilus*. For both of these bacteria, a significant decrease in binding was observed by 24 h (*p* ≤ 0.001). While the observed decreases may be the result of viral detachment from the bacteria, given the dramatically different growth conditions for *L. acidophilus* and *B. dorei* compared to the Proteobacteria, we questioned whether decreased attachment may be due to bacterial lysis rather than detachment, particularly because the bacteria are pelleted and washed prior to viral detection and lysis of bacterial cells would result in loss to the supernatant. The viability of the bacteria under our attachment assay conditions was tested over 24 h. Both *B. dorei* and *L. acidophilus* concentrations declined significantly over 24 h, often dropping below the limit of detection, whereas concentrations of *E. cloacae* and *E. coli* remained stable over the same time course (Figure S2). It should be noted that the lower levels of attachment to *L. acidophilus* observed after 1 h of incubation (Figure 1 and Figure 3), were not due to decreased bacterial survival (Figure S2). Loss of virus stability could also account for decreased genome detection, although severe reductions in genome copies were not observed for all bacteria (Figure 3). We quantified MNV concentrations in the absence of virus over 24 h under attachment assay conditions and found genome copies to remain relatively stable (Figure S3). Thus, loss of virus stability also does not likely explain the significant decreases in genome copies observed in *L. acidophilus* and *B. dorei* samples (Figure 3). After discovering the instability of *L. acidophilus* over 24 h, *L. gasseri* was not included in the time course experiments. Based on all these results, it is unknown how MNV attachment to *B. dorei* and *L. acidophilus* changes over time and further testing is needed to make this determination. 

### 3.3. Impact of Temperature and Bacterial Growth Phase on Murine Norovirus Attachment 

**Figure 4:**
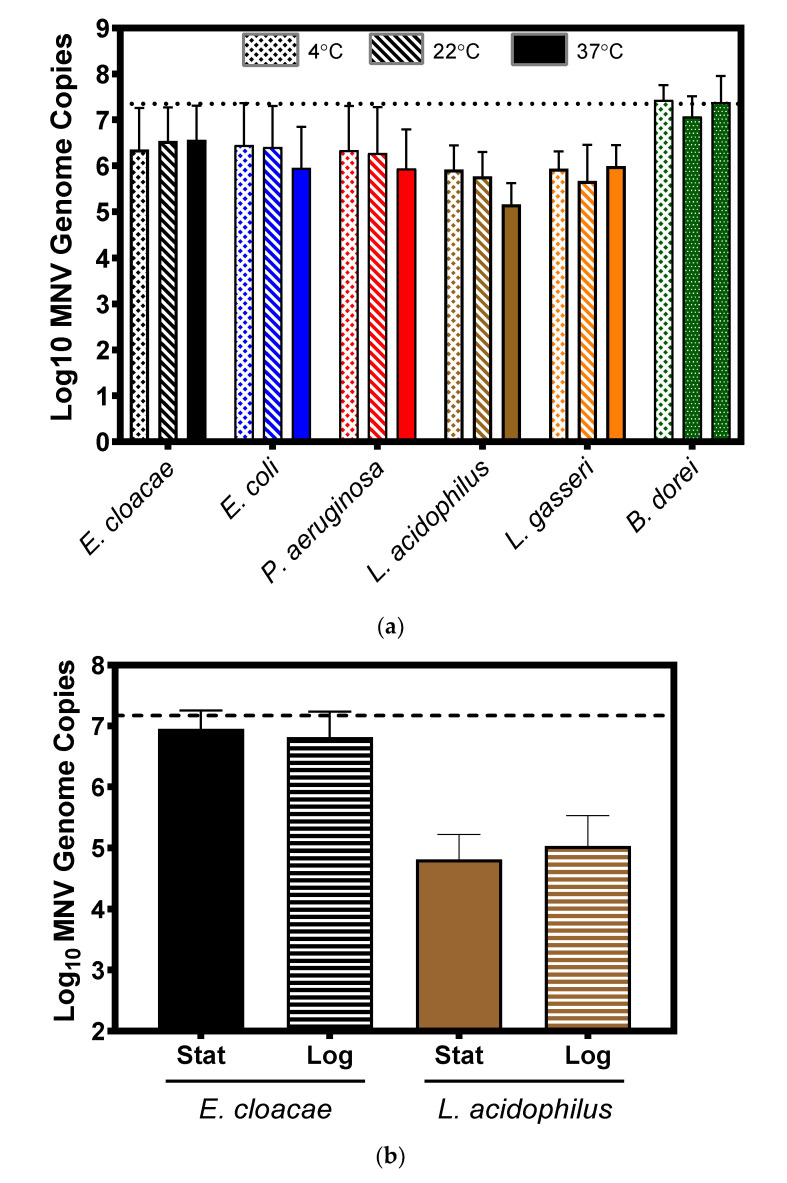
Impact of thermal incubation conditions and bacterial growth phase on murine norovirus attachment to bacteria. (**a**) Changes in incubation temperature (4, 22 and 37 °C) did not significantly alter MNV attachment to any of the tested bacteria. Statistical analysis was performed using a paired Student’s *t* test in GraphPad Prism. (**b**) Bacterial growth phase—log or stationary—did not significantly (*p* > 0.05) alter MNV attachment to either *E. cloacae* or *L. acidophilus*. The attachment was determined by the amount of viral genome copies found by RT-PCR. For all experiments and treatments, *n* = 3. Statistical analysis was performed using a paired Student’s *t* test in GraphPad Prism.

Growth and incubation conditions can impact expression of bacterial surface structure, particularly appendages such as pili and flagellum [28,29,30]. Therefore, we questioned whether incubating conditions under which the structures are altered would also alter MNV attachment to bacteria. To test the impact of temperature, MNV was incubated with the panel of bacteria for one hour at 4 °C, 22 °C, and 37 °C, in parallel. Results showed that for all bacteria there were no significant differences in viral attachment among the temperatures tested (Figure 4a). To investigate the impact of the bacterial growth phase on viral attachment, a representative gram-negative and gram-positive bacterium (*E. cloacae* and *L. acidophilus*, respectively) were chosen to determine if bacterial growth phase altered norovirus binding to bacteria. Attachment assays using stationary and log phase cultures were performed in parallel, but significant differences in attachment were not observed for either bacterium (Figure 4b). While these results do not conclusively determine bacterial surface structures bound by MNV, they indicate that this virus binds to structures that are consistently expressed under varying incubation conditions (e.g., outer membrane proteins) rather than to structures whose expression varies (e.g., pili and flagella). This observation is also supported by our electron microscopy data (Figure 1) which showed the virus bound to the outer membrane of the bacterium and not to any of the appendage-like structures. 

### 3.4. Murine Norovirus Attachment to Candida albicans

**Figure 5:**
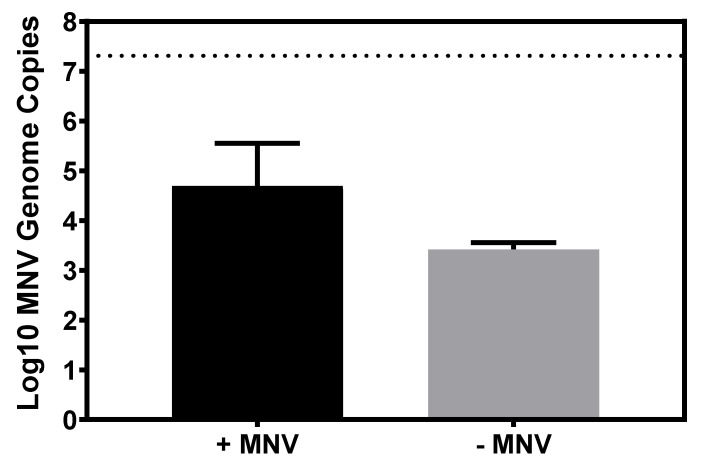
MNV attachment to *C. albicans*. RT-PCR showed that MNV genomes were detected in *C. albicans* pellets after 1 h of incubation (*n* = 3), while *C. albicans* pellets without MNV remained at the limit of detection (log_10_ 3.3 MNV genome copies). Dashed line represents amount of input virus. Statistical analysis was performed using a paired Student’s *t* test in GraphPad Prism.

Studies have indicated that bacteria make-up approximately 99% of the intestinal microbiome, with fungal and archaeal diversity making up the remaining 1% [31]. However, only a small fraction of the microbiome literature has been devoted to investigating the fungal communities within the microbiome, leading to the assertion that fungal diversity and numbers are subject to high fluctuations and may at times comprise larger or smaller percentages than what has been previously measured [32]. Based on the presence of fungi in the mammalian gut and the ability of MNV as well as other enteric viruses to bind to many commensal bacteria, the ability of MNV to bind to the common commensal fungal species, *C. albicans*, was investigated. While the levels of viral binding to *C. albicans* tended to be lower than what was observed of bacteria, MNV clearly attached to the fungi (Figure 5). 

## 4. Discussion

Previous work has demonstrated that human noroviruses bind to commensal bacteria regardless of viral or bacterial strain [24]. However, the ability of the commonly used surrogate, MNV, to bind to bacteria has not yet been explored. Given that this virus is commonly used to elucidate mechanisms of pathogenesis for extrapolation to human norovirus, it is important to understand if both viruses interact with commensal bacteria in a similar fashion. To answer this issue, we quantified viral binding and bacterial population binding in parallel for a murine and human norovirus, respectively. Our results demonstrated that not only is MNV capable of directly binding to the enteric bacterium *E. cloacae*, but that it also attaches to other bacteria commonly found in the mammalian intestinal microbiome. For both HNoV and MNV, less attachment was observed with *L. acidophilus*. However, studies examining bacterial survival indicate that post-attachment processing may lead to reduced viability of the bacterium, which could lead to a loss of virus and thus be responsible for the reduced genome copy numbers observed for MNV. Our HNoV VLP results are also consistent with those previously published where the majority of the viral population binds to *E. cloacae* and other bacteria within 1 h [24]. It has been demonstrated that HNoVs in stool and HNoV VLPs likely bind to Histo-blood group antigen (HBGA)-like residues found within the extracellular polysaccharide of commensal bacteria and electron microscopy images have demonstrated the ability of the virus to bind proteinaceous structures as well [23,24,26]. For MNV, the bacterial surface structure(s) bound by the virus are currently unknown. Given that HBGAs are not needed for host infection for MNV as they are for HNoV, it may be that MNV binds to different bacterial structures. During infection, MNV utilizes terminal sialic acids on gangliosides of macrophages [33]. Interestingly, mammalian commensal bacteria are hypothesized (and in some case have been demonstrated) to express sialic acid sugars within their capsular polysaccharides [34,35], possibly as a form of host mimicry. Thus, it may be that, in the same way that HNoV binds to the host-like HBGA carbohydrates on the surface of commensal bacteria, MNV may bind to host-like sialic acids on the same bacteria. In addition, sialic acids have a diverse array of sub-types and configurations that may allow for increased expression on the surface of bacterial cells and this may contribute to increased binding on some bacteria and decreased binding for other species. 

In addition to characterizing attachment to bacterial species, we set to determine if MNV can attach to other inhabitants of the gut microbiota, specifically *C. albicans*. While levels of viral attachment to *C. albicans* were lower than the commensal bacteria tested, the virus still bound to the commensal fungus to a slight degree. The ratio of input virus to bound virus was approximately 100:1, meaning that only ~1% of the virus remained attached to the fungal surface. It is currently unknown what structure(s) MNV was bound to on *C. albicans* or what effects this attachment has on infection for either organism. Though the outer structures of commensal fungi and bacteria differ greatly, the former is encased in a cell wall made of repeating glycans and the latter is surrounded in either an outer membrane or a thick peptidoglycan layer, they still share expression of certain carbohydrates on their surfaces [36]. The cell wall of *C. albicans* is dynamic and can change in response to the environmental conditions of the fungus as it can grow as a single-celled yeast, pseudohyphae, or true hyphae form [37]. These readily reversible forms serve as virulence factors, however, our culture conditions specifically allowed for growth of primarily yeast cells. These cell walls are composed of a variety of glucans, chitin, and glycoproteins and they have been shown to express sialic acids on the terminal ends of their glycan chains [38]. As sialic acids are a diverse group of ligands, it is possible that the structure bound on bacteria by MNV is similar in composition to the structures bound on *C. albicans,* but this relationship will need to be explored in further studies.

Since norovirus transverses the intestine and is exposed to commensal bacteria for longer periods of time, we next evaluated if attachment of MNV to commensal bacteria changed over time. Interestingly, while binding to *E. cloacae* and *P. aeruginosa* were steady over 24 h, binding to *E. coli*, *L. acidophilus*, and *B. dorei* all declined over the same time period. For *L. acidophilus* and *B. dorei*, the viability of the bacteria decreased over time, which may have impacted xviral attachment. On the other hand, *E. coli* concentrations remained stable, indicating that MNV was detached and that viral kinetics may differ between our *E. coli* strain and other similar bacteria, such as *E. cloacae*. The reasons for this are not yet known, but these observations led us to hypothesize that MNV may bind to specific surface structures and binding would change as expression of these structures changed. In fact, it has been previously observed that human norovirus binding to bacteria changes with incubation in different bacterial growth medium [24]. We therefore tested MNV binding to bacteria under conditions that are known to alter the expression of structures previously shown to be bound by human noroviruses. Unfortunately, results from these experiments did not support this hypothesis, as significant differences in MNV attachment were not observed among the varied conditions. It should be noted that expression of bacterial surface structures were not quantified and it may be that their expression was not sufficiently changed to alter viral attachment. Specific surface structure binding and the impact in changes in structure expression on viral binding are currently being explored to address this issue.

In conclusion, these experiments demonstrate that, like other enteric viruses, murine norovirus is capable of directly binding to commensal bacteria. Viral attachment varies among bacterial strains and the kinetics of attachment may also differ, even among closely related bacterial species. Given that the mechanism of bacterial enhancement of MNV infection is not yet known, this work will aid in better defining norovirus–bacterial interactions and in elucidating the role the microbiome plays in MNV pathogenesis.

## Supplementary Materials

The following are available online at https://www.mdpi.com/1999-4915/12/7/759/s1. Figure S1: Survival of bacteria in the presence of MNV, Figure S2: Survival of MNV over time, Figure S3: Growth phases of *C. albicans*.
