# Supplementary material for: Attach Me If You Can: Murine Norovirus Binds to Commensal Bacteria and Fungi
Source: Viruses. 2020 Jul 14;12(7):759. doi: 10.3390/v12070759 (PMC7412252; doi:10.3390/v12070759)
Supplement: Supplementary file 1 [file viruses-12-00759-s001.pdf]

viruses

Article

Attach Me If You Can: Murine Norovirus Binds to Commensal Bacteria and Fungi

Jasmine L. Madrigal, Sutonuka Bhar, Samantha Hackett, Haley Engelken, Ross Joseph, Nemat O. Keyhani and Melissa K. Jones *

Microbiology and Cell Science Department, IFAS, University of Florida, Gainesville, FL 32611, USA
* Correspondence: mmk@ufl.edu

Received: 02 May 2020; Accepted: 08 July 2020; Published: 14 July 2020

Supplemental Materials and Methods

Enumeration of bacteria in the presence of MNV. Attachment assays between virus and bacteria were performed as previously described for time course assays. All sample preprations were performed within a BSL-2 hood. At each time point an aliquot of virus:bacteria was spun down at 10,000 x g for 5 min, washed twice with equal volume of 1 x PBS, then finally resuspended in 1 mL 1 x PBS. Samples were serially diluted from 10^1 to 10^6 then each dilution was spot plated onto appropriate agar plates in triplicate, with each spot containing 10 µl of sample. Agar plates were left to dry without lids for ~10 min then incubated with lids and in a non-inverted position, with the agar side facing up, under appropriate conditions. For *Lactobacillus acidophilus* and *Bacteroides dorei* plates were incubated in anaerobic chambers at 37 ˚C for two days while *Escherichia coli* and *Enterobacter cloacae* plates were incubated aerobically at 37 ˚C for 1 day. After incubation, bacterial colonies were counted for each time point.

MNV stability over time. MNV-1 was inoculated into PBS at a concentration of 1 x 10^7 $TCID_{50}$/mL and incubated at 37 °C with rotation for 24 hrs in the manner performed for virus-bacteria attachment assays. Samples were taken at the indicated time points, RNA extracted and RT-qPCR performed as outlined in the manuscript to determine MNV genome copies.

Optimization of *Candida. albicans* growth. Light microscopy of cells from solid culture was used to further confirm cell morphology. Based on the parameters examined above, *C. albicans* was cultured on Sabouraud Dextrose Agar (SDA) plates with pH 5.6 +/- 0.2 at 25 ˚C for regular strain maintenance in the laboratory. Under these conditions, cultures were observed to consist almost entirely of yeast-like cells in fresh cultures. To prepare liquid cultures for use in viral attachment assays, isolated colonies from three-day old SDA cultures of *C. albicans* were picked with toothpicks and inoculated into 5 mL of Sabouraud Dextrose Broth (SDB) (pH 5.6 +/- 0.2) and incubated overnight at 25 ˚C with shaking at 200 rpm.

Supplemental Results

Determination of bacterial survival. Survival of bacterial strains included in MNV and HNoV attachment assays were assessed over time. *E. coli* and *E. cloacae* concentrations remained stable, but *B. dorei* and *L. acidophilus* concentrations declined and often dropped below the limit of detection by 24 hrs.

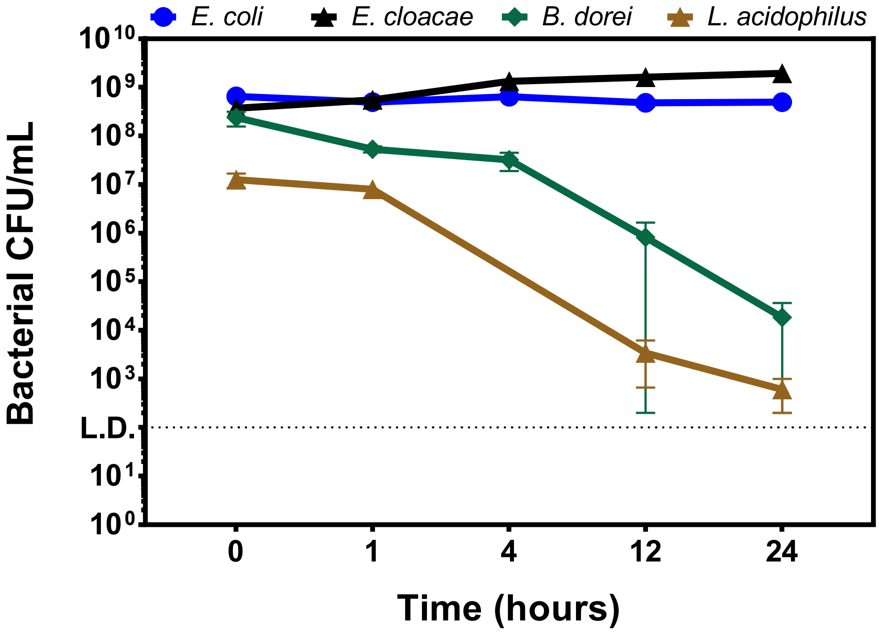

Figure S1. Survival of bacteria in the presence of MNV. Survival of representative bacteria over 24 hrs was tested in the presence of murine norovirus. Both *E. cloacae* and *E. coli* concentrations remain stable over time. However, *B. dorei* and *L. acidophilus* concentrations decline rapidly after 1-4 hrs of incubation. Significance over time was determined used a one way ANOVA and differences at given time points between specific samples was analyzed using unpair Students t test, both in GraphPad Prism.

Determination of MNV-1 survival. Survival of MNV-1 in the absence of bacteria was measured over time under conditions used in virus-bacterial attachment assays. Results showed MNV genome copies remained relatively stable over 24 hrs and no significant decreases in virus concentration was observed compared to amounts of input virus.

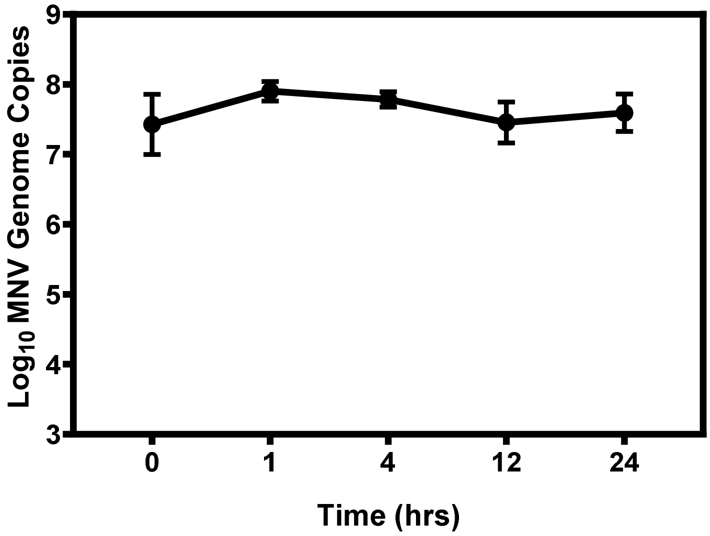

Figure S2. Survival of MNV over time. Survival of MNV at 37 °C was assessed over 24 hrs using conditions that mimicked virus-bacteria attachment assays. RT-qPCR was used to quantify viral genomes at the given time points. No significant decreases in virus concentration were observed (n=3). Significance over time was determined using a one sample t and Wilcoxon test in GraphPad Prism.

Optimization of C. albicans growth. As the goal of this study was to asses viral attachment to C. albicans cells under conditions of commensal growth, it was important to ensure that C. albicans was reliably cultured in the yeast-like state. To this end, culture conditions were optimized by addressing growth medium, pH, growth temperature, and culture age. For these optimizations, colony morphology was used as a readout for cell morphology, as yeast-like cells appear smooth in culture, whereas hyphae appear wrinkled (Figure S1). The optimum growth conditions for generating yeast-like cells was determined to be growth in SDB broth and overnight (~18 hrs) incubation at 25 ˚C.

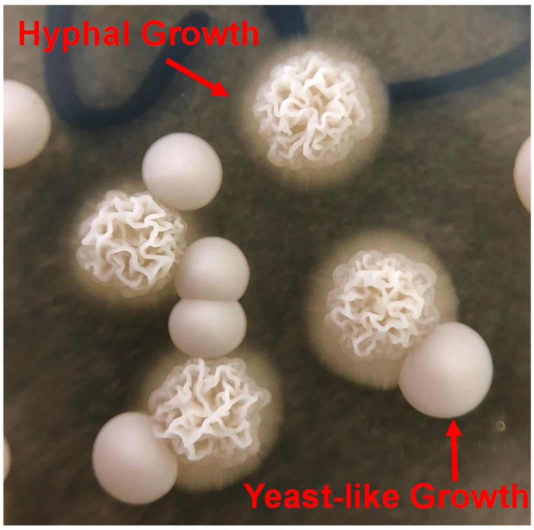

Figure S3. Growth phases of *C. albicans*. Light microscopy reveals the hyphal and yeast-like growth phases of *C. albicans*. Fungal growth conditions were optimized for generation of the yeast-like form for inclusion in MNV attachment assays.
